# Validation of triggers and development of a pediatric trigger tool to identify adverse events

**DOI:** 10.1186/s12913-014-0655-5

**Published:** 2014-12-21

**Authors:** Maria Unbeck, Synnöve Lindemalm, Per Nydert, Britt-Marie Ygge, Urban Nylén, Carina Berglund, Karin Pukk Härenstam

**Affiliations:** Department of Orthopedics, Danderyd Hospital, 182 88 Stockholm, Sweden; Department of Clinical Sciences, Danderyd Hospital, Karolinska Institutet, 182 88 Stockholm, Sweden; Division of Pediatrics, Astrid Lindgren’s Children’s Hospital, Karolinska University Hospital, 171 76 Stockholm, Sweden; Department of Clinical Science, Intervention and Technology, Karolinska Institutet, 171 77 Stockholm, Sweden; Department of Women’s and Children’s Health, Karolinska Institutet, 171 77 Stockholm, Sweden; Unit for Quality and Patient Safety, Karolinska University Hospital, 171 76 Stockholm, Sweden; SALAR (Swedish Association of Local Authorities and Regions), 118 82 Stockholm, Sweden; Medical Management Centre, Karolinska Institutet, 171 77 Stockholm, Sweden

**Keywords:** Adverse event, Trigger tool, Retrospective record review, Pediatric care, Safety

## Abstract

**Background:**

Little is known about adverse events (AEs) in pediatric patients. Record review is a common methodology for identifying AEs, but in pediatrics the record review tools generally have limited focus. The aim of the present study was to develop a broadly applicable record review tool to identify AEs in pediatric inpatients.

**Methods:**

Using a broad literature review and expert opinion with a modified Delphi process, a pediatric trigger tool with 88 triggers, definitions, and descriptions including AE preventability decision support was developed and tested in a random sample of 600 hospitalized pediatric patients admitted in 2010 to a single university children’s hospital. Four registered nurse-physician teams performed complete two-stage retrospective reviews of 150 records each from either neonatal, surgical/orthopedic, medicine, or emergency medicine units.

**Results:**

Registered nurse review identified 296 of 600 records with triggers indicating potential AEs. Records (*n* = 121) with only false positive triggers not indicating any potential AEs were not forwarded to the next review stage. On subsequent physician review, 204 (34.0%) of patients were found to have had 563 AEs, range 1–27 AEs/patient. A total of 442 preventable AEs were found in 161 patients (26.8%), range 1–22. Overall, triggers were found 3,598 times in 417 (69.5%) records, with a mean of 6 (median 1, range 0–176) triggers per patient. The overall positive predictive value of the triggers was 22.9%, (range 0.0-100.0%). The final pediatric trigger tool, developed with a second Delphi round, required 29 triggers.

**Conclusions:**

AEs are common in pediatric patients and most are preventable. The main contributions of this study are to further develop and adapt trigger definitions, including AE preventability decision support, to introduce new triggers in pediatric care, as well as to apply pediatric triggers in different clinical specialties. Our findings resulted in a national pediatric trigger tool, and might also be adapted internationally. The pediatric trigger tool can help healthcare organizations to measure and analyze the AEs occurring in hospitalized children in order to improve patient safety.

## Background

The challenge for healthcare organizations to learn from adverse events (AEs) remains. In order to provide healthcare teams with an adequate picture of potential AEs within their system, different means of identifying and analyzing AEs have been developed. Structured retrospective record review of admissions is an established method for identifying AEs that often go unnoticed by using incident reporting systems [1-5]. Published AE incidences in children range from 1% to 62% of the admissions in pediatric care [6-12].

One of the most used retrospective record review methods is the Global Trigger Tool (GTT), developed by the Institute for Healthcare Improvement in the USA, which has been used for diverse adult patient populations [13]. Kirkendall et al. [7] used the GTT developed for adult care on a pediatric sample, and found that 25.8% of the patients had at least one AE. This study showed the potential use for the GTT in pediatric populations, but it also pointed out that the review process needs to be developed since definitions and reference values in existing tools are not adapted to pediatric care. Development of such definitions has been done for some clinical contexts, and trigger tools have been developed, for example, for neonatal intensive care, pediatric intensive care, critically ill children, otolaryngology, and for the detecting of adverse drug events (ADEs) [6,8,14-17], but there is limited knowledge about overall AEs in pediatric care. A challenge to the practical use of the GTT in patient safety management in pediatric care has been the absence of a single tool suitable for examining AEs across pediatric populations and events. A promising comprehensive trigger tool for hospitalized children in Canada, based on the Harvard medical practice study review methodology, found AEs in 15.1% [10] and 9.2% [9] of the admissions. Another pediatric trigger tool was launched in the United Kingdom in 2010 and a recently published study reported that at least one AE occurred in 14.2% of the patients [18]. Both these studies represent a development of trigger tools for detecting AEs in a pediatric population. However, the findings still point to a need for further development of the this methodology [18] and if the aim is to promote learning from AEs, continuous development of triggers, trigger definitions and the framework for categorizing AEs is needed.

All acute care hospitals for adult patients in Sweden perform monthly record reviews and enter the results into a national database according to a financial incentive provided by the government. The need for the development of a national tool for detection of AEs in pediatric populations was identified.

The aim of the present study was to develop a broadly applicable national pediatric trigger tool (PTT) including a manual with a thorough description of triggers, including definitions, reference values, AE preventability decision support, and a description of the review methodology.

## Methods

**Figure 1:**
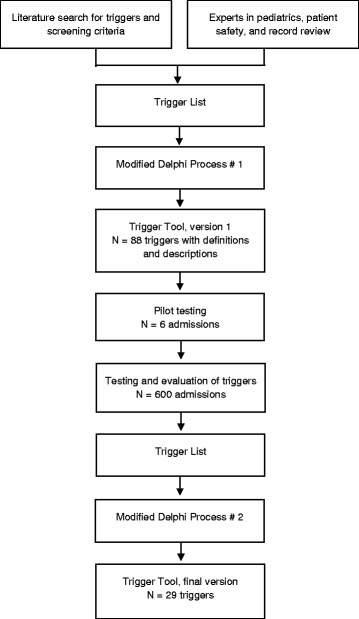
**Flowchart of the development of the pediatric trigger tool.**

The overall PTT project was initiated from a university hospital and the Swedish Pediatric Society with the aim of developing a tool suitable for manual trigger search in pediatric care. A national expert group of 20 persons (Figure 1) was established from different subspecialties within the Swedish Pediatric Society, clinical advisors from other professions, as well as researchers to serve as a reference group.

The PTT development was based on previous studies using record review tools with triggers or screening criteria. To collect information on existing tools, the research group conducted a literature review and developed a study design. PubMed© was searched using the keywords trigger tool, adverse event, harm, medical record, record review, reporting systems, pediatric, children, adult, safety management, inpatient and/or outpatient. Twenty different record review tools for manual use available in English were identified. Nine of the tools were tested in specific pediatric specialties, pediatric units, or types of AEs; two of the latter included AEs from several pediatric specialties. Two trigger tools, not available via PubMed©, was also included. A preliminary set of triggers and screening criteria from different record review methods and studies were added to a list, with duplicated or similar triggers/screening criteria removed. After this, additional recommended triggers later approved by the reference group were added. The first trigger list, including a proposal of definitions and descriptions, was sent by e-mail to the reference group for discussions at the respective pediatric units before a one-day face-to-face meeting. Using a modified Delphi process [19], the reference group was asked to add, change, and remove triggers while also adding relevant pediatric reference values to the triggers in addition to changing the trigger definitions and descriptions. The triggers were discussed and evaluated based on their clinical relevance (i.e., trigger face validity or pointing at high-risk/high-volume AEs) and utility (i.e., usefulness for quality improvement and local ability to act on the results). This process resulted in a pediatric trigger tool (PTT) consisting of 88 triggers (Figure 1) in seven modules (*Care, Surgical, Medication, Intensive care, Infant, Perinatal,* and *Emergency Department modules*). As none of the existing record review tools met our requirements, our aim was to make sure that the final PTT was inclusive for AEs in different types of pediatric care. Therefore, the study deliberately included a broad range of well defined triggers based on clinical and research experiences, as well as on the literature review and record review experiences, with the aim of validating these in a patient sample.

## Test of the pediatric trigger tool

The PTT was tested and later evaluated at an urban children’s hospital in Sweden, one of seven divisions at a university hospital located at three different sites and consisting of 19 units. The children’s hospital generally admits patients from 0 to 18 years of age and has eight departments with different specialties such as surgery, orthopedic, oncology, intensive care, neonatology, and medicine, for example. At the time of the study, the children’s hospital had a capacity of 250 beds, had around 2,000 employees, and provided care for approximately 25% of all children in Sweden. Records of children under 19 years receiving all levels of inpatient care discharged during 2010 with at least a 24-hour length of stay (study population *n* = 12,765) were eligible for randomization. The final cohort consisted of 600 admissions (4.7% of all admissions), and was selected by randomization into four blocks of 150 admissions from neonatal, surgical/orthopedic, medicine, and emergency medicine units, respectively. One review team for each block consisting of one registered nurse (RN) and one physician was formed. The study sample reflected 5,559 hospital days for index admissions, comprised a majority of male patients (*n* = 314, 52.3%), and acutely admitted patients (*n* = 474, 79.0%). The mean age was 4.3 years (SD 5.3 years, median 18 months, range 0–18 years), and mean length of stay was 9.3 days (SD 16.6, median 4, range 1–138). The youngest patient was born in gestational week 24 plus one day.

### Definitions and inclusion criteria

An AE was defined as an unintended harm to the patient caused by health care rather than by the patient’s underlying disease process [20]. Harm means physical harm, as psychological harm is difficult to identify in record review studies. The severity of harm can be anywhere between minor, such as a transient allergic reaction to a drug, or major such as permanent disability or death. Both acts of omission and acts of commission were included. A preventable AE was defined as an AE which could has been prevented if adequate actions had been taken during the patient’s contact with health care [21].

The pediatric admission in the random sample of 600 admissions during 2010 constituted the index admission. To be included as an AE in the study, one of the following three criteria had to be met: (1) The AE occurred within 30 days before index admission, caused the index admission, or was detected during the index admission; (2) the AE occurred and was detected during index admission; (3) the AE occurred during index admission and was detected within 30 days of index discharge from the children’s hospital. Adverse events identified by using the latter criterion were not required to result in a new admission; in other words, an AE treated on an outpatient basis was included.

### Review teams, training and pilot testing

The four review teams consisted of RNs with great experience within pediatric care and in the respective specialty; three out of four physicians were consultants in neonatal, medicine, and surgical care, respectively. All of the RNs as well as two of the senior physicians were unfamiliar with the GTT method, yet they were chosen because of their interest as well as the hospital wanting to collect internal knowledge about the method for future work with quality improvement.

The review teams were, besides the reference group, involved in the development of trigger definitions and descriptions, as well as manual development. To standardize the review process in order to achieve valid and reliable outcomes, a written manual, as a complement to the Swedish manual in the GTT methodology [22], was developed, discussed, and approved by the reference group and all reviewers before the study start. A one-day education in the GTT methodology was performed for all review team members. During the process of familiarization with the methodology and as a pilot test of the triggers and the manual, each review team member independently reviewed six test/training records before a consensus process allowed discussions of the interpretation and application of the triggers, further refinement of definitions as well as the manual, AE assessments, and related matters (Figure 1). An example of the description of a trigger used in the study is shown in Table 1. The 88 triggers and trigger names were not changed after this process. Strategy discussions were carried out concerning how to make the review process more reliable and efficient. During the study period, support was available via reconciliation meetings, e-mail, as well as telephone access primarily with one of the investigators (MU).

**Table 1:** **Example of a trigger description**

**Urinary retention**	
*Definition*	Urinary retention (age and weight related)
	10 ml per kilo (children up to 20 kg) + 20%
	100 ml (children ≥ 20 kilo) + (age in years x 20) + 20%
*Description of the trigger*	Urinary retention is to be assessed as an adverse event if there is more urine in the bladder than listed in the values above. Patients who have urinary retention on admission will be excluded unless it can be considered to derive from earlier treatment within 30 days.
	Urinary retention can occur, for example, in connection with pain, opiate treatment, epidural anesthesia, or spinal cord compression.
	Inter individual variations concerning the maximum volume of the bladder exist.
	Be observant of urine amounts in connection with, for example, surgery and analgesic, and review the monitoring curves per- and postoperatively, and the nursing documentation.
*Harm that can be detected*	Risk of bladder muscle damage due to over tension can cause the patient pain, discomfort and urinary tract infection. Bladder muscle harm can be permanent if over tension becomes severe or lasts for a long time. For the patient, this can mean lifelong needs of mechanical emptying of the bladder.
	Risk of bladder muscle damage due to over tension can cause the patient pain, discomfort, and urinary tract infection. Bladder muscle harm can be permanent if over tension becomes severe or lasts for a long time. For the patient, this can mean lifelong needs of mechanical emptying of the bladder.
*Preventability*	These adverse events should be assessed as preventable.
*Drugs (ATC), diagnosis (ICD-10) or procedure code associated with this trigger*	ICD-10-code:
	R33.9 (urinary retention)
*Measures or products associated with this trigger*	Urinary retention for children, age- and weight-related. Detected by, for example, bladder scan.

A database, with access only for the study members, developed in Microsoft Access© 2007 Microsoft Corporation US, with case report forms supporting the two review stages was used throughout the study. The review teams were given instructions both verbally and written on how to use the database.

### The two-stage review process

In Review Stage 1, all records in electronic format were reviewed by the respective RN, one for each group. The RNs screened for the presence of one or more of the 88 predefined triggers. For every trigger detected, a judgment was made by the respective RN regarding whether the trigger reflected the presence of a potential AE or not. The RNs documented how many times the respective trigger were present per patient as well as the time taken to review each record. Only records with triggers indicating at least one potential AE were marked by the RNs in the database to be reviewed by the physicians. Records not containing any triggers or containing only false positive triggers, i.e. triggers not indicating AEs were not forwarded to physicians for review but the outcome was documented and included in the analysis. The RNs also recorded demographics data, and entered all patients into the database. No time restriction existed in this review stage.

In Review Stage 2, the physicians performed an independent review of the records with at least one potential AE forwarded from the RNs in Review Stage 1. The physicians sorted the different triggers into potential AEs and every potential AE were reviewed separately. To qualify as an AE in the physician review, more than 50% likelihood of healthcare causation, in other words, a score of four or higher using a 6-point scale, must have been present [23,24]. A similar 6-point scale was used to judge the preventability of the AE. A score of four or more meant that the AE was deemed to be preventable [24]. The severity of the AE was judged using an adaptation of the National Coordinating Council for Medication Error Reporting and Prevention (NCC MERP) Index [25]. The NCC MERP Index categories E-I were included, in other words, those relating to harm. All physicians documented all triggers related to each AE due to the fact that an AE could have been identified by ≥1 trigger. The reviewers in Stage 2 documented the review time.

### Reliability and validity

The nurse review process was evaluated. First, every tenth record was double reviewed to assess agreement between the RNs’ judgments. The RNs could not see the other RNs’ judgments in the database. After independent review of all records in each group, the RNs discussed the duplicate reviewed records and reached consensus. The consensus results were forwarded to the respective physician for review. The RNs in the neonatal and surgery/orthopedic teams double reviewed together, as did the RNs in the medicine and emergency medicine teams. Second, in Review Stage 2 the physicians, while performing their independent review, included any additional AE they found that had not been identified by the RNs in Stage 1.

All reviews from Stages 1 and 2 in the database were monitored by a record review expert (MU) searching for completeness, and all questions or discrepancies were referred back to the respective reviewer for resolution. In Review Stage 2, no double review was performed. The record review expert compared the physicians’ review outcome with the study manual, including methodology, and had clarifying discussions with the respective physician if discrepancies were found.

## Ethical approval

Ethics approval was provided by the regional Ethics Committee of Stockholm (number 2012/2014-31/5). Permission for data collection for patients, triggers, and AEs through the electronic patient record system was granted by the head of the children’s hospital and by each department chairman.

## Statistics

Categorical data are summarized using frequency counts and percent. Continuous variables are presented as mean with standard deviation (SD) and median with range. Both the number of AEs per record and the number of records with at least one AE are presented.

Positive predictive value (PPV) of the respective trigger was counted as the number of times a specific trigger identified an AE divided by the total number of times the trigger was found. A trigger identified several times in a single patient in Review Stage 1 became additional cases; for instance, five transfusions to the same patient resulted in five triggers and were classified as C1-Transfusion. Positive predictive values (PPV) over 100% due to physicians identifying additional triggers to the AE not documented in the RNs’ reviews are presented as 100%.

The statistical programs used to collate the results were QlikView 11 by Qlik Technologies, Inc. PA, US, and Stata 12.0 by StataCorp, TX, US.

## Results

No records were excluded in the study due to missing documentation.

In Review Stage 1, after excluding 121 records with only false positive triggers, 296 (49.3%) of the 600 records were forwarded to physician review. The physicians identified 1,066 potential AEs from Review Stage 1. After Review Stage 2, 563 different AEs were identified in 204 (34.0%) patients, range 1–27. Of these, 442 AEs (78.5%) in 161 (26.8%) patients, range 1–22, were deemed to be preventable.

The RNs verbally evaluated that the PTT was easy to use, but reported some difficulties in applying a few trigger definitions such as for pain, for example. The proportion agreement between the RNs concerning: identification of the exact same triggers in the same records were 46.1%, identification of records containing triggers were 65.0%, and records containing at least one potential AE were 75.0%. Of the 15 records where the RNs did not agree that the records should be reviewed by a physician, eight records did not contain an AE according to the physician. The remaining seven records included one minor AE each. There was a 68.9% agreement between RNs’ and physicians’ regarding the existence of at least one AE in the record. Half (50.5%) of the 1066 potential AEs, identified via triggers in Review Stage 1, were confirmed as AEs by the physicians. In their independent review of the records containing at least one potential AE, the physicians found a total 24 AEs not identified by the RNs.

The mean time for the review process was 23.9 minutes (median 15, range 2–250) in Review Stage 1 and 17.8 minutes (median 7, range 1–298) in Review Stage 2.

## Trigger outcome

Triggers were totally identified 3,598 times in 417 (69.5%) records, resulting in a mean of 6.0 (SD 15.6) triggers per patient (median 1, range 0–176). There were 109 records with one trigger, and 220 records had five to nine triggers. Individual triggers varied widely in their yield of detections of AEs after Review Stage 2 (PPV range 0.0-100.0%), and the overall PPV of the triggers was 22.9% (Table 2). The PPV for records containing at least one trigger identified by the RNs and an AE confirmed by the physicians was 48.9% (204/417). The PPV was low in the most frequently identified triggers, such as *Transfusion* (4.55%) and *Failures in cardiovascular, respiratory or neurological function* (4.1%). More patient records were found to be trigger-positive in neonatal, surgery/orthopedic, and medicine units vs. the emergency medicine units corresponding to the lower AE incidence in these units. The PPV were: for the neonatal, 24.1%; surgical/orthopedic, 21.0%; medicine, 26.2%; and emergency medicine, 10.2%. A wide variability in the number of trigger outcome and number of AEs associated with the respective trigger was observed within the seven modules. The *Intensive care* and *Surgical modules* were the most predictive ones, and the *Infant* and *Perinatal modules* the least predictive. Most AEs were identified in the largest modules; *Care* and *Medication*.

**Table 2:** **Outcome of respective trigger in relation to the adverse event sorted by positive predictive value**

**Original triggers N = 88**	**n (%) of patients with ≥ 1 of the respective trigger**	**n of triggers detected by RNs**	**n of triggers related to AE**	**PPV, %**	**Revised triggers N = 29** ^**a**^
Infiltration/extravasation of intravenous injection/ infusion	40 (6.7)	60	71	100.0	Blood vessel, skin and/or tissue harm
Pressure ulcers	9 (1.5)	14	18	100.0	Blood vessel, skin and/or tissue harm
Urinary retention	9 (1.5)	10	10	100.0	Urinary retention
Positive culture from central line catheter or insertion site	9 (1.5)	10	10	100,0	Positive culture
Clostridium difficile positive stool	5 (0.8)	5	5	100.0	Hospital-acquired infection
Neurological harm	4 (0.7)	5	6	100.0	Neurological impairment/harm
Anesthesia related harm	4 (0.7)	4	4	100.0	Anesthesia-related impairment/harm
Anaphylactic reaction	2 (0.3)	2	3	100,0	Anaphylactic reaction
Ventilator-associated pneumonia	2 (0.3)	2	2	100.0	Ventilator-associated pneumonia
Hallucinations/delirium/ICU syndrome	1 (0.2)	1	1	100.0	Other
Occurrence of any postoperative complication	17 (2.8)	20	19	95.0	Postoperative impairment/harm
Abnormal body temperature	16 (2.7)	19	17	89.5	Abnormal body temperature
Post-operative infection	11 (1.8)	14	12	85.7	Hospital-acquired infection
Other side-effect of drug	34 (5.7)	36	27	75.0	ADE/ADR
Readmission to the Intensive Care Unit	4 (0.7)	4	3	75.0	Unplanned transfer to higher level of care
Skin- and blood vessel harm, thrombophlebitis	100 (16.7)	242	168	69.4	Blood vessel, skin and/or tissue harm
Positive blood culture	30 (5.0)	40	27	67.5	Positive culture
Unplanned drug withdrawal	4 (0.7)	3	2	66.7	ADE/ADR
Change in procedure or technique	7 (1.2)	7	4	57.1	Change in procedure/organ harm
Intubation/reintubation/tracheotomy/ coniotomy	25 (4.2)	36	20	55.6	Intubation, tracheotomy, or coniotomy
Dissatisfaction with care	14 (2.3)	13	7	53.8	Documentation of mistake or dissatisfaction with care
Occurrence of mistake	77 (12.8)	94	50	53.2	Documentation of mistake or dissatisfaction with care
Fungal infection	27 (4.5)	30	15	50.0	Hospital-acquired infection
Reoperation	12 (2.0)	18	9	50.0	Reoperation
Instrumental delivery	16 (2.7)	14	7	50.0	Other
Unplanned removal of and/or harm of an organ during surgery or other invasive action	5 (0.8)	6	3	50.0	Change in procedure/organ harm
Unplanned mechanical ventilation greater than 24 h post-operatively	2 (0.3)	2	1	50.0	Cardiac arrest or failures in vital signs
Naloxone administration	2 (0.3)	2	1	50.0	ADE/ADR
Wound rupture	2 (0.3)	2	1	50.0	Blood vessel, skin and/or tissue harm
Rising serum creatinine	6 (1.0)	10	4	40.0	Renal impairment/harm
Readmission to the ED within 48 hours	4 (0.7)	3	1	33.3	Unplanned readmission within 30 days
Unplanned dialysis	2 (0.3)	3	1	33.3	Renal impairment/harm
Other infection	79 (13.2)	117	38	32.5	Hospital-acquired infection
Aspiration	14 (2.3)	11	3	27.3	Cardiac arrest or failures in vital signs
Pain	127 (21.2)	234	62	26.5	Pain
Unplanned intubation/reintubation/ delayed extubation/CPAP/BiPaP	7 (1.2)	8	2	25.0	Cardiac arrest or failures in vital signs
Apgar < 7	17 (2.8)	17	4	23.5	Decreased vitality in infant
Pneumonia	17 (2.8)	17	4	23.5	Hospital-acquired infection
Readmission within 30 days	81 (13.5)	92	21	22.8	Unplanned readmission within 30 days
Necrotizing enterocolitis	7 (1.2)	9	2	22.2	Necrotizing enterocolitis
Antidote administration	8 (1.3)	14	3	21.4	ADE/ADR
Care: other	71 (11.8)	114	24	21.1	Other
Transfer to higher level of care	28 (4.7)	34	7	20.6	Unplanned transfer to higher level of care
Operative time greater than 6 h	5 (0.8)	5	1	20.0	Other
Antibiotic treated urinary tract infection	16 (2.7)	16	3	18.8	Hospital-acquired infection
Pathological blood gas from umbilical cord blood	12 (2.0)	12	2	16.7	Decreased vitality in infant
Time in ED greater than 6 hours	6 (1.0)	6	1	16.7	Other
Post-operative admission to intensive care unit	6 (1.0)	6	1	16.7	Unplanned transfer to higher level of care
Ultrasound guided drainage	4 (0.7)	6	1	16.7	Hospital-acquired infection
Partial Thromboplastin Time (PTT) greater than 100 seconds	2 (0.3)	7	1	14.3	ADE/ADR
Any codes or arrest	34 (5.7)	96	13	13.5	Cardiac arrest or failures in vital signs
C-reactive protein > 200 mg/liter	25 (4.2)	62	7	11.3	Hospital-acquired infection
Viral gastroenteritis	32 (5.3)	53	6	11.3	Hospital-acquired infection
Abnormal liver enzymes	23 (3.8)	45	4	8.9	ADE/ADR
Abnormal potassium value	18 (3.0)	48	4	8.3	ADE/ADR
Glucose < than 3 mmol/liter or administration of 300 mg/ml or 500 mg/ml glucose	54 (9.0)	154	12	7.8	Hypoglycemia
Vitamin K administration (excluding newborns)	15 (2.5)	44	3	6.8	ADE/ADR
Neutropenia and antibiotic treatment	13 (2.2)	65	4	6.2	Hospital-acquired infection
Abnormal sodium value	17 (2.8)	36	2	5.6	ADE/ADR
Too high or too low drug concentration	14 (2.3)	40	2	5,0	ADE/ADR
Transfusion	72 (12.0)	400	18	4.5	Cardiac arrest or failures in vital signs
Failures in cardiovascular, respiratory or neurological function	114 (19.0)	778	32	4.1	Cardiac arrest or failures in vital signs
Induced delivery	88 (14.7)	90	3	3.3	Other
Abrupt drop in hemoglobin	46 (7.7)	111	3	2.7	Cardiac arrest or failures in vital signs
Thrombocytes < 50 x109/liter	20 (3.3)	87	2	2.3	ADE/ADR
11 triggers not related to an AE^b^	28 (4.7)	33	0	0.0	
**Total**	**429 (71.5)**	**3598**	**824**	**22.9**	

RN, registered nurse; AE, adverse events; PPV, positive predictive value; ICU, intensive care unit; ADE, adverse drug event; ADR, adverse drug reaction.

^a^The revised trigger column includes the triggers in the final trigger list. For the removed triggers an example of a trigger in the final trigger list suitable for the detection of corresponding AEs is given.

None of the triggers *Deep vein thrombosis or embolism, Positive culture from cerebrospinal fluid, Intra- or postoperative death, Post-operative increase in troponin levels, Wrong site/wrong procedure/wrong patient, Flumazenil administration, Sodium polystyrene administration, Interactions, Transfer of mother/child, Terbutaline administration, or 3rd- or 4th-degree lacerations* were identified in this study.

^b^None of the triggers: *International Normalized Ratio (INR) greater > 5, Glucose > 20 mmol/liter, Activation of dose range checking, Intensive care unit procedure, Ultrasound of the brain > week 32 - < 3 months, In-hospital stroke, Falls, Intra-operative administration of administration of inotropes/antidotes, Abnormal pathology report, Unplanned insertion of arterial or central venous line during surgery, Post-operative pleural fluid* were associated with an AE in Review Stage 2.

## Development of the final trigger list and the design of the PTT

After analysis and discussions of the triggers, PPVs, and AE outcomes, a third trigger list was created and followed by a second Delphi process round (Figure 1). During the end of the study period, the national adult GTT manual and method had been revised/further developed in parallel. When deciding about which triggers would be included in the final trigger list, the revised adult triggers were taken into account so the two trigger tools were harmonized, as far as possible, with regard to trigger names and the ability of the tool to also include no-harm incidents [26].

After evaluation of the outcome and the review process, the number of pediatric triggers was reduced in order to be convenient for use in a nationally applied clinical record audit manual. Triggers with low PPV, which were inexplicit or had low clinical relevance in pediatric care, were removed. For example, the triggers *Abrupt drop in hemoglobin* and *Transfusion* were removed from the trigger list, as corresponding AEs will be detected by triggers such as *Cardiac arrest or failures in vital signs* or *Reoperation* in the final trigger list.

Triggers that could be merged were merged into a wider trigger to reduce the number of triggers, as where eleven medication triggers were merged into one implicit trigger, for example. Some of the trigger definitions and descriptions were refined with the aim of achieving a more valid PTT, thus reducing the false positive trigger outcomes as with, for example, a low glucose value in newborns needing to occur more than once to be included as a positive trigger. Three new triggers were added after the analysis of the results: *Loss of weight, Extreme hyperbilirubinemia* and *Severe retinopathy of prematurity*. The set of triggers and definitions was edited and refined until consensus was reached (Figure 1). The *Emergency Department module* was removed, a *Laboratory module* was added and the *Perinatal* and *Infant modules* were merged into one.

The resulting national PTT consists of 29 triggers in six modules (Table 3), and includes a manual with thorough description of the respective trigger, AE preventability decision support, and the review process. A further mapping on trigger level was conducted to assure that the old list of triggers was consistent and covered by the new set of triggers in the final trigger list (Table 2).

**Table 3:** **Final trigger list consisting of 29 triggers in six modules**

**Modules**	**Triggers**
*Care module*	Stroke
	Cardiac arrest or failures in vital signs
	Deep vein thrombosis or pulmonary embolism
	Blood vessel, skin and/or tissue harm
	Neurological impairment/harm
	Abnormal body temperature
	Hospital-acquired infection
	Unplanned transfer to higher level of care
	Documentation of mistake or dissatisfaction with care
	Pain
	Unplanned readmission within 30 days (including outpatient visits)
	Loss of weight
	Urinary retention
	Other, not covered by any other trigger
*Laboratory module*	Hypoglycemia
	Renal impairment/harm
	Extreme hyperbilirubinemia
	Positive culture
*Surgical and invasive procedure module*	Reoperation
	Change in procedure/organ harm
	Postoperative impairment/harm
	Anesthesia-related impairment/harm
*Medication module*	Anaphylactic reaction
	Adverse drug event/adverse drug reaction
*Intensive care module*	Ventilator-associated pneumonia
	Intubation, tracheotomy or coniotomy
*Infant module*	Decreased vitality in infant
	Necrotizing enterocolitis
	Severe retinopathy of prematurity

## Discussion

To our knowledge, this is the largest record review study testing a manual trigger tool method for inpatients in a single children’s hospital. A total of 563 AEs in hospitalized children of all ages and in several specialties constituted rich material for validation of the triggers and led to the design of a PTT with 29 triggers that will be used as a national tool for manual record review checking for quality and safety management.

There are several methodological considerations when continuing the development of trigger tool methodology that we want to raise.

### Comparison between different trigger tools is cumbersome

When interpreting the validity of a trigger tool, both information about the number of times a trigger is detected and the percentage of patients behind a trigger’s PPV is important. The PPV for records containing at least one trigger identified by the RNs and an AE confirmed by the physicians was in our study 48.9%. Our overall PPV for the trigger outcome of the PTT was 22.8%, while other trigger tools in pediatric care range from 3.75% to 44% [6,15-18,27]. Making comparison between trigger tools may, in some cases, be problematic due to that the respective tool’s overall PPV [7,8,10,18,28-31], the actual number of times a trigger occurs in the different review stages, or the number of patients per trigger [6,8,10,16,17,27] are not reported. The overall PPV of a trigger tool is influenced by the interpretation of the trigger definitions, the number of triggers included, and how multiple occurrences of the same trigger have been dealt with. Our study design depicted that every time a trigger was identified, it should be counted, and this resulted in lower PPVs for certain triggers. That in turn affected the overall PPV of the PTT. For example, a hemorrhage leading to five *Transfusions* was documented five times in Review Stage 1, but was then documented as one trigger related to one AE in Review Stage 2. In hindsight, the physicians in Review Stage 2 should have reported the triggers in the same way as RNs in Review Stage 1. The emergency medicine units yielded the lowest PPV and AE outcome. This might reflect the care given in these settings, reflecting non-invasive treatments like inhalations and intravenous or oral rehydration in children without previous or chronic illness, meaning, therefore, that they are not exposed to the same risk as compared to units such as surgery or neonatal care.

### There is variability in the outcomes of different studies

We identified more triggers per patient (mean 6.0 triggers per patient) compared to other pediatric studies searching for AEs or ADEs, where results ranged from 1.65 to 2.96 triggers per patient [7,16-18,27]. The PPV of the triggers in our study had a wide variability in coherence with others [7,10,16], and the PPV for specific triggers, for example, the trigger *Any code or arrest* was, in some cases, reversed compared to other studies: 13.5% in our study vs. 50% or 62.5% in others [7,10]. Some of the triggers such as *Transfusion, Abrupt drop in hemoglobin, Pain, Glucose <3 mmol/l or administration of 300 mg/ml or 500 mg/ml glucose and Failures in cardiovascular, respiratory,* and *neurological function*, for example, were common in Review Stage 1, but were often judged to be false positive in the RN and physician reviews. Some of the triggers with low PPV and inexplicit definitions in the study have still been included in the final trigger list after clarification of the definitions, as they represent important aspects of quality in pediatric care such as *Pain*, for example.

### There are several possible factors influencing the information yield from a record review and the variability in AE outcome

We found that one-third of the patients were affected by AEs with a wide range of AEs per patient. Other pediatric studies have identified AEs in 1% to 62% of admissions [6-12]. One factor explaining the AE incidence is how an AE is defined. The AE definition used in the present study was inclusive, in accordance with the GTT methodology, and did not require that the patient should have experienced a disability or prolonged hospital stay which is the definition of AEs that originates from the Harvard medical practice study methodology [23]. The use of this narrow definition probably explains the lower AE incidence reported in earlier pediatric studies [9-12]. This was highlighted in one pediatric study [10], where 340 injuries were identified in 180 records, but only 89 records met the inclusion criteria for an AE.

Another factor influencing the information yield is variability in reviewers’ judgments. This includes reviewer skills, application of triggers and definitions, experience and prevalent views on patient safety of the reviewers, and training and education in the methodology [32,33]. Over half of the potential AEs were rejected as being AEs in the physician review. This may reflect the record review experience of the RNs as well as the review manual dictating that any unclear events should be forwarded to the physician for review. As a way to further develop the review methodology, we chose to refrain from having a second secondary reviewer, and instead developed a detailed manual and process for crosschecking with the manual in Review Stages 1 and 2 by using a review expert.

### Considerations while developing the PTT

Our final PTT consists of several implicit triggers, for example, several medication triggers were merged into one wider trigger to increase the usability for manual review. The use of such implicit triggers is similar to- the Harvard medical practice study methodology’s where 18 rather implicit screening criteria are used. Future development of trigger tools will have an important methodological choice between developing tools that are easy to use for manual review with implicit triggers in contrast to explicit and detailed triggers that meet the requirements needed for computerized data mining tools.

As our knowledge of the nature of AEs in different care settings continues to grow new triggers emerge. The importance have been raised of capturing previously unclassified triggers by having a trigger named *Other* as a part of further trigger tool development based on review experience [7]. An example of such a trigger in Swedish healthcare is *Urinary Retention* in adult care that was found to occur in 9.1% of all AEs in acute care hospitals [34]. This trigger, not found in any other record review tool, had a PPV value of 100% in our study. Analysis of the outcome of the trigger *Other* is also important to identify rare types of AEs. The absence of a trigger of this kind has been reported to cause underestimation of ADEs or AEs in up to 16.8% [6,17].

The final trigger list was constructed in accordance with the methodological developments in the Swedish adult GTT [26], making it possible to include no-harm incidents (NCC MERP Index categories C and D) [25]. This is in line with the conclusions of an earlier study [35] showing that retrospective record review methodology is suitable for identifying this type of safety information while searching the record for AEs in order to inform proactive patient safety management.

## Strengths and limitations

The strengths in our study are the sample size in one single children’s hospital with several specialties and that the reviews followed the patients both in inpatient and outpatient care. We have also made an effort to transparently describe the development of the triggers and the review process. There are several limitations in the present study. The most known limitations belong to the method itself, requiring documentation quality in the records, otherwise leading to a risk of underestimation of the numbers of AEs, in addition to the lack of a golden standard for AE identification with which we can compare our results. However, several studies have shown that retrospective record review is a superior method of identifying AEs compared to other methods, such as clinical incident reports [1-5], for example.

The review was performed by newly trained team members, with the exception of two physicians who had previous experience identifying AEs using record review methodology. This was a given constraint in the study design since the method has not yet been used in pediatric care in Sweden, and pediatric knowledge was an inclusion criterion for the reviewers. It was also a request from the hospital that the study would help develop a group with methodology knowledge that could continue working with record review as one part of the hospital’s patient safety management.

Regardless which retrospective record review is used, the methodology is subjective and can be affected by certain biases such as with the hindsight bias, which may provide an overestimation, especially of the determination of healthcare causation, preventability, and severity of an AE. We tried to standardize the application and interpretation of the PTT by having teams involved in the project phase. This included a detailed study manual with specific instructions, definitions and trigger descriptions, regular meetings during the study period, access to the investigators for questions, and a review expert who monitored the study database and gave the reviewers regular feedback.

In our study design the physicians did not review records negative for potential AEs leading to that Cohen’s kappa between physician and RN review could not be calculated. We therefore only reported percent agreement for both review stages.

Another limitation is that we did not validate the final trigger list on an additional sample of records or reanalyzed the reviewed records. This is because the final list of triggers was an outcome of the study and we chose in the design of the study to include a broad range of triggers thus primarily aiming to validate individual triggers. However, a comprehensive analysis was made to assure that the new trigger list did indeed cover the individual triggers validated in the 600 records. A further validation of the final trigger list will be performed in clinical practice when the PTT is in use in pediatric care nationally.

The study was limited to one children’s hospital; although the hospital provides approximate 25% of all pediatric care in Sweden, this may affect the ability to generalize the outcome.

## Conclusions

This study develops the trigger tool methodology for pediatric care in a study conducted in a large children’s hospital setting covering a wide range of care. The main contributions of this study were to further develop and adapt trigger definitions, AE preventability decision support, introduce new triggers in pediatric care, and to apply pediatric triggers in different clinical specialties. Retrospective review methodology is an important source of safety information, and further development and adaptation of the method is important in order to provide clinicians and health care providers with adequate information about the gaps and risks in healthcare systems, as well as allow for further research on the epidemiology of pediatric iatrogenic harm. Our findings resulted in a national PTT which might also be adapted internationally.

## Abbreviations

ADE: Adverse drug event
AE: Adverse event
GTT: Global Trigger Tool
NCC MERP: National Coordinating Council for Medication Error Reporting and Prevention
PPV: Positive predictive value
PTT: Pediatric trigger tool
RN: Registered nurse
